# Transforming Smiles: A Case Study on Monolithic Zirconia Prosthetic Solutions

**DOI:** 10.7759/cureus.57889

**Published:** 2024-04-09

**Authors:** Mithilesh M Dhamande, Arushi Beri, Seema Sathe, Tanvi Jaiswal, Surekha A Dubey

**Affiliations:** 1 Prosthodontics and Crown & Bridge, Sharad Pawar Dental College, Wardha, IND; 2 Prosthodontics, Sharad Pawar Dental College and Hospital, Acharya Vinoba Bhave Rural Hospital (AVBRH), Wardha, IND; 3 Prosthodontics, Sharad Pawar Dental College, Wardha, IND; 4 Prosthodontics and Crown & Bridge, Sharad Pawar Dental College and Hospital, Datta Meghe Institute of Medical Sciences (Deemed to be University) (DMIMSU), Wardha, IND

**Keywords:** cad cam, smile correction, anterior aesthetics, monolithic zirconia, transforming smiles

## Abstract

In order to effectively address challenges related to anterior teeth restoration and achieve natural-looking results, considerations such as shape, size, gingival contour, and color are crucial. Due to an increasing desire for visually appealing alternatives that are free of metal, materials such as dental zirconia have become popular because of their superior aesthetics and mechanical characteristics. This case report presents clinical insights into anterior teeth rehabilitation with the use of layered zirconia fixed dental prostheses. It delves into the experiences associated with zirconia dental restorations on both endodontically treated and vital abutments, aiming to discern how various factors influence treatment outcomes. Beginning with the design of the restoration, its intricacies significantly impact its fit, strength, and overall durability. Moreover, the composition of zirconia used plays a pivotal role, as different formulations offer varying degrees of mechanical properties, influencing factors such as resilience and wear resistance. The shade selection is also scrutinized, as it directly affects the restoration's aesthetic integration with surrounding natural teeth, contributing to a more harmonious smile. Furthermore, the layering technique employed, particularly when additional porcelain or ceramic layers are applied, is essential for both cosmetic enhancement and structural integrity. Lastly, considerations of occlusion are paramount, ensuring proper alignment and contact between teeth to prevent premature wear and discomfort. By exploring these facets in zirconia restorations across different abutment types, this inquiry seeks to illuminate best practices for achieving favorable treatment outcomes in dental restoration procedures. The choice of zirconia composition, framework design, and shade must be carefully tailored to suit the characteristics of each individual abutment. This emphasizes the significance of adopting a tailored approach to tackle the distinct challenges posed by every clinical scenario.

The manuscript provides detailed observations from a clinical case involving the restoration of anterior teeth utilizing monolithic zirconia-fixed dental prostheses. Through a combination of root canal treatment and composite buildup, successful restoration was achieved, with meticulous attention paid to aesthetic considerations. The utilization of computer-aided designing/computer-aided manufacturing (CAD/CAM) technology in crafting zirconia restorations ensured precise fit and superior biocompatibility, contributing to the overall success of the treatment. The study underscores the importance of personalized treatment strategies in achieving optimal outcomes in anterior teeth restoration, emphasizing the need for careful consideration of various factors such as design, composition, and shade selection. Overall, the findings shed light on the potential of zirconia-based restorations in addressing the unique challenges associated with anterior teeth rehabilitation, offering valuable insights for dental practitioners striving to deliver aesthetically pleasing and functionally sound outcomes for their patients.

## Introduction

Restorative dentistry places a strong emphasis on achieving aesthetic perfection, as patients often seek dental treatments not only to restore functionality but also to enhance the appearance of their smiles. Dental ceramics, particularly zirconia, have long been favored in this field for their exceptional properties and ability to mimic the natural appearance of teeth. Zirconia crowns, especially those with a full contour design, have gained immense popularity, particularly in restoring maxillary anterior teeth where both aesthetic and functional outcomes are crucial. This paper presents a detailed case study illustrating the treatment of a patient using zirconia all-ceramic crowns. The results not only meet stringent aesthetic standards but also ensure optimal functional performance, significantly enhancing the patient's psychological well-being [1-4].

It is essential for dentists crafting ceramic crowns to be highly attuned to patients' aesthetic preferences. Merely focusing on technical accuracy may not suffice to fulfill individual desires for symmetry and aesthetics. Achieving successful results hinges on thoroughly grasping patient expectations and carefully considering anatomical aspects. The case study also explores the use of computer-aided designing/computer-aided manufacturing (CAD/CAM) zirconia material, particularly in cases of extensive dental attrition. This exploration highlights the growing importance of zirconia in modern dental procedures, showcasing its versatility and effectiveness in addressing complex dental issues [5].

This case study provides valuable clinical observations regarding the restoration of anterior teeth using layered zirconia fixed dental prostheses. It explores the challenges and successes encountered in treating both endodontically treated and vital abutments, considering various factors including prosthesis design, zirconia material properties, shade matching, layering methods, and occlusal considerations, and their effects on treatment results. Overall, the study contributes to advancing the understanding and practice of restorative dentistry, offering valuable insights for dental practitioners and potentially enhancing patient satisfaction and treatment outcomes [6-10].

## Case presentation

**Figure 1:**
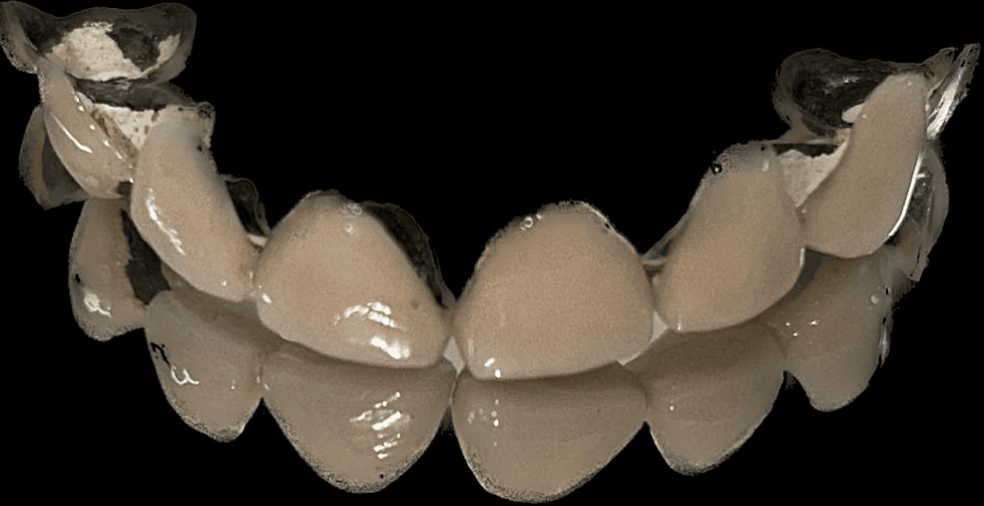
Worn off porcelain fused to metal crowns

The scenario described pertains to a 42-year-old woman who is looking for a long-lasting cosmetic fix for the deterioration of porcelain-fused-to-metal crowns on her upper front teeth (Figure 1).

A comprehensive assessment was undertaken, involving a thorough review of the patient's medical history and detailed examinations. These examinations revealed extensive discoloration of all the teeth which may be indicative of fluorosis and previous porcelain-fused-to-metal (PFM) restorations in the teeth. Vital pulp testing using electric pulp testing confirmed the need for root canal treatments with right and left central incisors, prompting discussions with the patient about various treatment options the lateral incisors and canine were vital. With the patient's consent obtained, a meticulous treatment plan was devised, focusing on using zirconia crowns for the upper front teeth. Intraoral examinations identified multiple composite restorations, while radiographic analysis showed signs of periodontal issues. Initially, impressions of the upper and lower teeth were taken, followed by scaling, polishing, and improving existing restorations. A collaborative effort with the dental technician resulted in a diagnostic wax-up, and composite resin was utilized to shape the upper incisors, selecting shade A1 to enhance aesthetics monolithic zirconia crowns were selected to mask the shade of teeth which were disolored. Tooth preparations were performed concurrently with elective root canal therapy.

**Figure 2:**
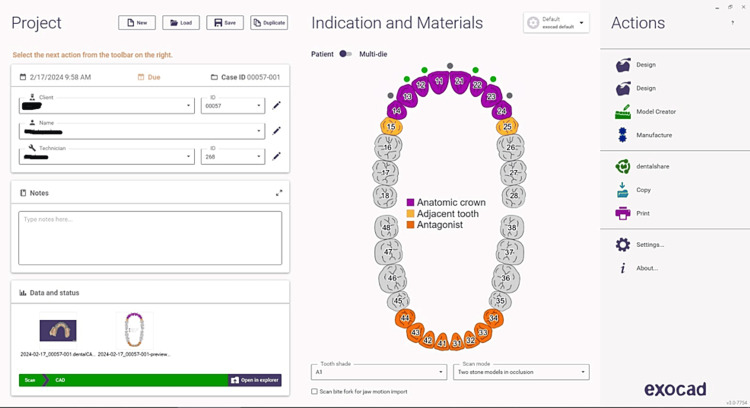
Designing using exocad software exocad DentalCAD 3.0 Galway, released in December 2020, is developed by exocad GmbH, a dental CAD/CAM software company based in Darmstadt, Germany.

**Figure 3:**
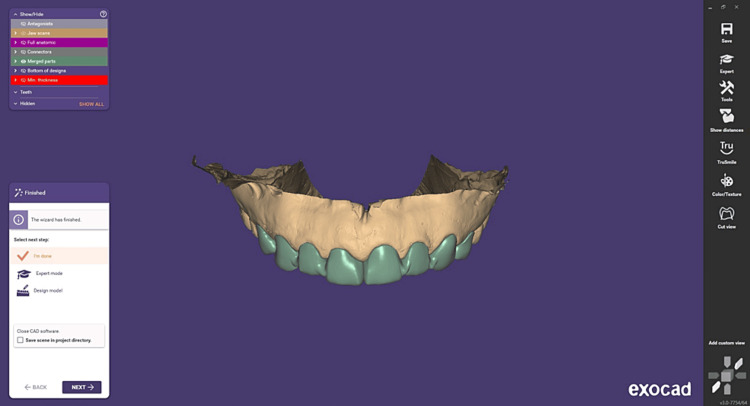
CAD of zirconia crowns 14-24 CAD - Computer-aided designing exocad DentalCAD 3.0 Galway, released in December 2020, is developed by exocad GmbH, a dental CAD/CAM software company based in Darmstadt, Germany.

To ensure accurate impressions, a retraction cord was placed carefully along the gum line to push the soft tissues away from the teeth. Full-arch impressions were then taken using polyvinyl siloxane material and the putty reline technique, capturing detailed impressions of the teeth and surrounding structures, the cast was then poured and scanned. These were sent to the dental laboratory, where CAD-CAM (exocad DentalCAD 3.0 Galway, released in December 2020, is developed by exocad GmbH, a dental CAD/CAM software company based in Darmstadt, Germany). FSZ Zirconia crowns were meticulously designed and fabricated to ensure a precise fit and optimal aesthetics using exocad software (Figures 2, 3).

During the final appointment, a porcelain try-in was performed, allowing the patient to preview the appearance of the crowns before final placement. Any necessary adjustments were made to ensure proper occlusion, ensuring that the teeth come together correctly when the patient bites down. After confirming the ideal fit and appearance, the zirconia crowns were glazed to enhance their aesthetics further.

**Figure 4:**
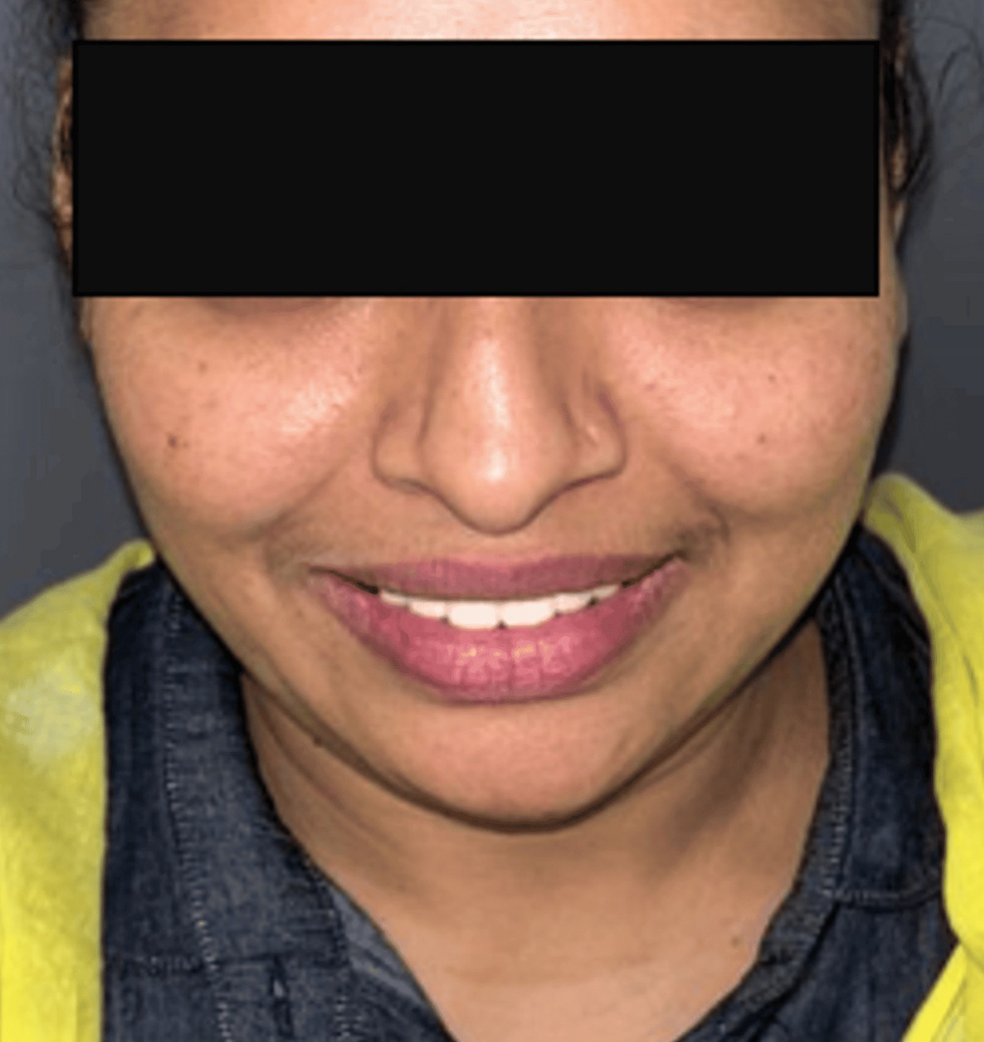
Post-op picture

All the instructions were followed as per the manufacturer's guidelines, the glazed zirconia crowns were carefully cemented into place on the prepared teeth. The post-operative outcome of the treatment was documented, likely through photographs or radiographs (Figure 4).

To ensure the treatment's long-term success, a follow-up protocol was implemented to evaluate the durability and efficacy of the zirconia crowns over time. This case underscores the critical role of thorough planning in achieving aesthetic and functional success in dental rehabilitation, stressing the significance of precise procedures and diligent monitoring for optimal results.

## Discussion

CAD/CAM zirconia ceramic prostheses possess remarkable characteristics resembling natural teeth, including a translucent quality, excellent compatibility with oral tissues, and enamel-like wear patterns. These features make them highly preferred, when matched to lithium silicate ceramics, although multifaceted they might lack comparable adaptability, particularly for more extensive restorations in the posterior part of the mouth. Our examination revealed that the most common Vertucci classification type in upper front teeth was type I, indicating a single canal with a single root and canal structure. However, we also identified variations beyond type I, complicating elective root canal procedures for these teeth [11-14].

Advancements in zirconia ceramics, specifically with fifth-generation mixed zirconias, have widened their range of applications and resulted in the development of specialized material variants. Fifth-generation mixed zirconia provides a wide range of applications, from small front-mouth bridges to larger bridges comprising up to 14 units, which allow for flexible use in dental restorations. A study of upper front teeth restored with CAD/CAM zirconia crowns after root canal treatment revealed a survival rate equal to or higher than alternative all-ceramic materials like PFM crowns and e.max ceramic, as verified by clinical and radiographic evaluations [15-17].

In this case, the patient experienced successful hiding of discoloration with durable and biocompatible crowns, leading to positive clinical results. The matching of color accurately addressed esthetic concerns along with it enhanced the patient's self-confidence and social interactions, particularly in their professional role as a schoolteacher. The natural alignment of the appearance and smile while speaking and smiling served to underscore the efficacy of treatment in attaining both functional and esthetic objectives. [18-20].

## Conclusions

In conclusion, this case study provides valuable insights into the restoration of front teeth affected by fractures and discoloration. Through a meticulous approach prioritizing the preservation of existing tooth structure, successful restoration was achieved via a combination of root canal treatment and composite buildup of the fractured tooth. Aesthetic considerations played a pivotal role in the treatment strategy, leading to the selection of an all-ceramic restoration, specifically a layered zirconia restoration.

Zirconia restorative prostheses, crafted using CAD/CAM technology, offer remarkable biocompatibility, resulting in minimal wear on adjacent teeth and ensuring better aesthetics with consistent color stability over time. These inherent qualities not only contribute to the restoration of oral function but also have a profound positive impact on patients' social lives, confidence, and self-esteem. Within the realm of prosthodontics, zirconia-based restorations exhibit significant potential due to their outstanding chemical, mechanical, and clinical capabilities. As such, embracing zirconia-based restorative options represents a promising avenue for achieving optimal outcomes in dental restoration, thereby enhancing both patient satisfaction and overall treatment success.
